# Everybody's Page

**Published:** 1891-08-08

**Authors:** 


### EVERYBODY'S PAGE.

DR. CHAMBARD HENON reports that he has administered chocolate tablets of kola nut in obstetrical cases with such success as to have prevented entirely all symptoms of syncope.

THE general public in this country are always slow to adopt any innovation, however many recommendations it may have. Cremation has been no exception to this rule, but it must be growing in public favour if it is true that existing facilities for its practice in Liverpool are not adequate to the demand, and that the building of a new and large crematorium there is contemplated.

SOME gentlemen have got themselves into trouble by an ingenious endeavour to evade the law against lotteries. Their method was, it is alleged, to sell cheap pastry containing threepenny pieces and other coins. If they were detected by means of an informer, it was really a piece of base ingratitude on the part of the latter. The happy surprise of discovering money in a plum tartlet should have added so much to the ordinary joy caused by the consumption of the delicacy as to have sealed the lips in the direction of secrecy even if it opened them wider in the direction of jam tarts.

THE purity or impurity of olive oil can, according to M. Brulle, be determined by a very simple test. By means of nitrate of silver, he claims that the presence of other oils can, after a little practice, be detected with ease. His method appears to be to use a solution of twenty-five parts of nitrate of silver in one thousand parts of ninety-five per cent. alcohol. Sixty-five parts of this he adds to fifty-two of the oil to be examined, then he places the tube containing the oil in a glass vessel filled with boiling water and watches the changes produced in the colour. If the oil is pure a beautiful green tint is produced.

IN a paper read before the New York Medical Association, Dr. J. Mount Bleyer advocates the use of the phonograph in medical science. As it can be made to record many of the symptoms usual in diseases of the respiratory organs in both normal and abnormal states, it can be used with advantage to record the voices of singers when in good voice, and by making the normal record a standard the voice can be compared in case of any ailment. In didactic teaching it would also be of value, and he suggests that "good records of specimen patients illustrating a certain cough—such as the whoop of whooping-cough, asthmatic cough, bronchitis, stenosis of the larynx, and in croup and diphtheria—would be of great benefit to students.

A HOMELY method of removing foreign substances from the noses of children is reported in the *New York Record*. Its chief charm is its simplicity. The operator first places a thin cloth over the child's mouth; he then presses close the nostril which does not contain the unwelcome lodger; the final step is to blow quickly and strongly two or three puffs into the child's mouth. Result—exit the lodger with hurried steps.

A FRIEND of ours who had just moved into a new house in the country was somewhat astonished and annoyed one evening by being called upon by a perfect stranger, who expressed his delight at seeing him in the neighbourhood with a warmth and stammering effusiveness which is only to be found in company with strong drink. The unwelcome guest was, however, got rid of, and next morning, having awaked to a consciousness of his better self, sent an apology for intruding when in an *abnormal* state.

THE revolver nuisance is bad enough in this country, but it seems to be aggravated a hundredfold in America, where toy pistols are in such favour with the budding youth of that country. It appears to be a holiday pastime with these young gentlemen to give work to the surgeons whose time has too often to be devoted to extracting pieces of metal caps, wadding, and powder from self-inflicted wounds, which are dangerous in inverse proportion to their size. Lockjaw and other unpleasant complications are frequent results of this practice.

A CONTRIBUTOR to the *New York Medical Record* is tempted to enquire into the alleged susceptibility of the Boston Constitution to minute doses of arsenic. The fate of twelve jurors at Boston impelled them to set themselves the conundrum—Do lobsters contain arsenic? These "good men and true" appear to have been poisoned recently by eating tinned lobster. The question to be determined was whether the Massachusetts lobster, in its natural state, contained the deadly poison, or is the arsenic to be looked for in the food and drink which the animal takes into its stomach. By one gentleman it was contended, that the *natural* bright red colour is such as can scarcely be obtained without the aid of arsenic. This almost convinced, till a gentleman from Denver humbly suggested that the natural hue of the Colorado lobster is green. Additional disturbing elements were introduced in the way of a satisfactory solution of the difficulty by a chemist giving evidence that he inadvertently let fall into the water where the lobsters are caught a specimen of wall paper. Some of the water will now have to be analysed in order to settle a question of such national importance!

